# Combining Wireless Technology and Behavioral Economics to Engage Patients (WiBEEP) with cardiometabolic disease: a pilot study

**DOI:** 10.1186/s40814-019-0395-8

**Published:** 2019-01-15

**Authors:** Edith Angellotti, John B. Wong, Ayal Pierce, Benjamin Hescott, Anastassios G. Pittas

**Affiliations:** 1 Division of Endocrinology, Diabetes and Metabolism, Tufts Medical Center, 800 Washington Street, #268, Boston, MA 02111 USA; 2 Division of Clinical Decision Making, Tufts Medical Center, 800 Washington Street, #302, Boston, MA 02111 USA; 3 Tufts University School of Medicine, 145 Harrison Ave, Boston, MA 02111 USA; 4 Northeastern University, 360 Huntington Ave, Boston, MA 02115 USA

**Keywords:** Text messages, Blood pressure, Diabetes, Diet, Exercise

## Abstract

**Background:**

The long-term management of cardiometabolic diseases, such as type 2 diabetes and hypertension, is complex and can be facilitated by supporting patient-directed behavioral changes. The concurrent application of wireless technology and personalized text messages (PTMs) based on behavioral economics in managing cardiometabolic diseases, although promising, has not been studied. The aim of this pilot study was to evaluate the feasibility and acceptability of the concurrent application of wireless home blood pressure (BP) monitoring (as an example of “automated hovering”) and PTMs (as an example of “nudging”) targeting pharmacotherapy and lifestyle habits in patients with cardiometabolic disease (type 2 diabetes and/or hypertension).

**Methods:**

The Wireless Technology and Behavioral Economics to Engage Patients (WiBEEP) with cardiometabolic disease study was a single-arm, open-label, 7-week-long pilot study in 12 patients (mean age 58.5 years) with access to a mobile phone. The study took place at Tufts Medical Center (Boston, MA) between March and September 2017. All patients received PTMs; nine patients received wireless home BP monitoring. At baseline, patients completed questionnaires to learn about their health goals and to assess medication adherence; at the end of week 7, all patients completed questionnaires to evaluate the feasibility and acceptability of the intervention and assess medication adherence. Hemoglobin A1c was ascertained from data collected during routine clinical care in 7 patients with available data.

**Results:**

The majority of patients reported the text messages to be easy to understand (88%) and appropriate in frequency (71%) and language (88%). All patients reported BP monitoring to be useful. Mean arterial pressure was lower at the end-of-study compared to baseline (− 3.4 mmHg [95% CI, − 5 to − 1.8]. Mean change in hemoglobin A1c was − 0.31% [95% CI, − 0.56 to − 0.06].

**Conclusions:**

Among patients with cardiometabolic disease, the combination of wireless BP monitoring and lifestyle-focused text messaging was feasible and acceptable. Larger studies will determine the long-term effectiveness of such an approach.

## Background

The rising prevalence of cardiometabolic diseases, such as type 2 diabetes and hypertension, challenges a U.S. healthcare system that was not designed for efficient management of chronic conditions. Effective long-term management of chronic diseases requires patients to adopt and sustain a healthy lifestyle in addition to taking prescribed medications. Patients with chronic diseases might spend only a few hours every year with a doctor or a nurse, but they spend about 5000 waking hours each year doing everything else—including deciding whether to take prescribed medications, deciding what to eat and drink, whether to smoke, whether to exercise and making other choices that can profoundly affect their health [1]. Despite the extensive availability of pharmacotherapies, nearly half of treated patients do not have adequate blood pressure (BP) or glycemic control [2, 3]. The main contributors to not reaching therapeutic targets are poor medication adherence, lack of patient engagement and therapeutic inertia [4, 5]. Traditional medical care (including “routine” visits) accounts for only ~ 10% of the total variance in outcomes for chronic conditions, while behavioral factors account for about 40% [6]. Accordingly, there is a growing interest in developing simple methods to support patients outside the traditional healthcare settings to enhance the adoption of healthy lifestyle and medication adherence.

Over the past 10 years, the growth in wireless connectivity, the increased recognition for individualized medicine and the need for disruptive innovation to optimize health care value supported the development of the mobile health (mHealth)—defined as medical and public health practice supported by mobile (wireless) devices. The utilization of mHealth technologies is a promising approach to transform care of chronic conditions and support patients outside the traditional healthcare settings [7, 8]. Adherence to such technologies may be enhanced by applying principles from behavioral economics, defined as a combination of conventional economic principles with psychology to explain human behavior. Two behavioral economic principles of particular relevance to mHealth interventions include “automated hovering” and “nudging.” The “automated hovering” (e.g., using wireless technology to monitor BP between clinic visits) can effectively fill the gap of those 5000 hours each year that patients spend away from the traditional health care setting. "Nudging" is the systematic and evidence-based development and implementation of nudges in creating behavior change (e.g., via text messages to influence predictable flaws in behavior) [1]. Behavioral economics to facilitate behavior change has also garnered substantial interest in healthcare delivery redesign but remains under-studied in type 2 diabetes [9–14]. The concurrent application of wireless technology and behavioral economic principles did not significantly improve medication adherence or vascular readmission outcomes after acute myocardial infarction [15], but has not been studied in chronic conditions such as type 2 diabetes and/or hypertension. The aim of this pilot study was to evaluate the feasibility and acceptability of combing wireless BP home monitoring (as an example of “automated hovering”) and text messaging (as an example of “nudging”) in patients with type 2 diabetes and/or hypertension.

## Methods

### Study design

The Wireless Technology and Behavioral Economics to Engage Patients (WiBEEP) with cardiometabolic disease study was a single-arm, open-label, 7-week-long pilot study. Its aim was to evaluate a strategy that included text messaging and wireless BP home monitoring targeting medication adherence and improved lifestyle habits (exercise and/or diet) in patients with cardiometabolic disease (type 2 diabetes and/or hypertension). All patients received automated personalized text messages (PTMs) and patients with hypertension also received wireless BP home monitoring. Participants attended only one in-person visit (baseline visit) plus two “virtual visits” (week 3 and week 7) if they qualified for wireless BP home monitoring. During the “virtual visits,” patients received an email with mean blood pressure values and, if necessary, a suggestion for a change in antihypertensive therapy (previously discussed with the patient’s primary care physician). During the baseline visit, once the informed consent form was signed, patients completed questionnaires to learn about their health goals (e.g., preventing stroke) and to assess medication adherence using the MMSA-8 Morisky scale [16]; patients and investigators jointly reviewed the data and collaboratively set goals for medication adherence, exercise, and diet.

### Participants and setting

Patients were recruited from primary care and endocrinology clinics at Tufts Medical Center (Boston, MA): all patients who were pre-identified by their physician as potentially eligible were mailed a study invitation letter. When interested patients called back, a member of the research team verified the inclusion criteria and, if eligibility was confirmed, scheduled the baseline visit. Inclusion criteria were (a) established type 2 diabetes based on ICD codes (managed without or with pharmacotherapy) or hypertension based on ICD codes (managed without or with pharmacotherapy), (b) use of mobile phone able to receive and send texts (for all patients), and (c) use of a “smart” phone (for patients with hypertension). Exclusion criteria included (a) uncontrolled hypertension (systolic BP > 180 mmHg or diastolic BP > 120 mmHg), (b) pregnancy (past 1 year by report), (c) intent to become pregnant in the next year, or (d) any unstable medical or psychiatric condition or (e) any other reason that, in the opinion of the investigators, would impede adherence with study procedures or hinder completion of the study or increase risk. The study took place between March and September 2017. The Tufts IRB approved the study, and all participants provided written informed consent.

### Intervention

#### Nudging

All patients received PTMs that were semi-customized with the content selected from a bank of short, actionable options providing patient-tailored advice and motivation towards healthy lifestyle changes (exercise and/or diet), and increased adherence to cardio-metabolic (i.e., diabetes, hypertension) medications. Prior studies have reported that interventions with personalized messages have a larger effect size [17]; therefore, certain elements of the texts were semi-personalized via a “mail-merge” function with the patient’s preferred name, providing reminders, encouragement and motivation to improve adherence to medication, increase exercise or improve diet, based on patient’s needs and preferences. The bank of messages was developed with input from investigators, and content was developed for 3 modules: exercise, diet, and medication adherence. Personalized text messages followed key insights from behavioral economics (e.g., limiting choice overload, setting goal gradients) and were personalized based on patient-specified personal health goals identified in the baseline questionnaire. Frequency (times per week) and timing (time of the day) of PTMs was determined by the patient. Some PTMs were merged with patient’s preferred names. Some PTMs were bi-directional requesting patients to respond to a question with a simple “yes” or “no”.Table 1 provides examples of messages.

**Table 1:** Examples of text messages used in the WiBEEP pilot study

Diet	
At your next meal, and every day, fill half of your plate with fruits and vegetables.	
At your next meal, stay away from processed (white) carbohydrates.	
Choose fish high in omega-3 fatty acids, like salmon, trout, and herring.	
Exercise	
Take the stairs. Your body and your brain will thank you!	
Do not forget to exercise today. Exercise lowers risk of stroke.	
Today, round up a friend or family member and walk. Team sport is fun!	
Medication adherence	
Hello [NAME], remember to take your medicines today at [TIME].	
Medications work best if taken at a particular time. Don't forget your medications today.	
Use a pillbox with compartments for each day: it prevents double doses.	

#### Automated hovering

In addition to texting, 9 patients with hypertension managed without (*n* = 1) or with (*n* = 8) pharmacotherapy received wireless BP home monitoring using an FDA-approved device (iHealth Labs, Inc.) connected via Bluetooth to the patient’s phone (or tablet). During the baseline visit, investigators demonstrated the use of the monitor to ensure that patients understood the procedure and provided written step-by-step instructions. Patients received a welcome email with a link to a login page to set up a unique username and password. This subgroup was asked to check BP measurements twice a day after 5 or more minutes of rest, comfortably seated in a quiet room with their feet touching the floor; results were automatically and wirelessly collected via the iHealth MyVitals App into a cloud-based portal (sherbit.com) accessible to study staff. Acceptability of the intervention components (wireless BP monitoring and text messaging), improvements in medication adherence (MMSA-8 Morisky), and BP (collected via the monitors) were assessed at the end of the study. In 7 patients, change in HbA1c was also ascertained from available data in electronic health records collected during routine clinical care.

## Results

**Fig. 1:**
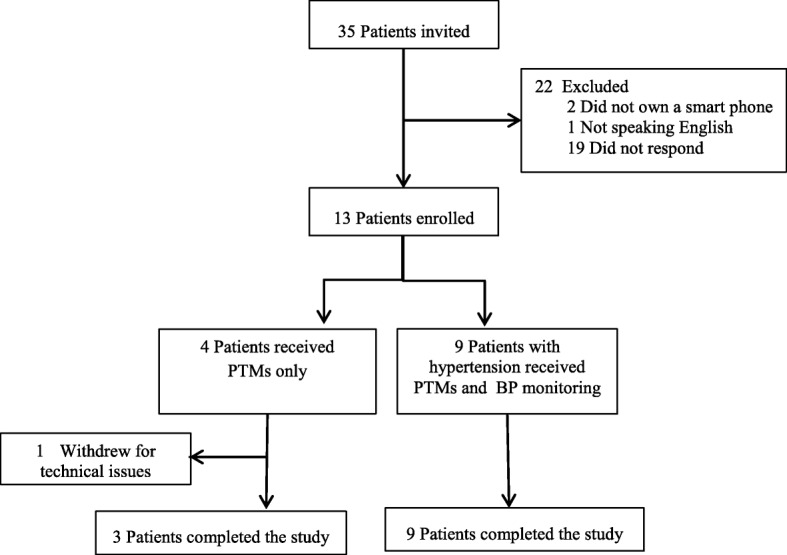
Flow diagram of participants. PTMs personalized text messages, BP blood pressure

In total, 35 patients were invited to participate in the pilot study. Of those, 13 were eligible and 12 completed the study (Fig. 1). Mean age of patients was 58.5 years (range 41 to 71), and BMI was 31 kg/m^2^. Twenty-five percent did not have a college degree (Table 2).

**Table 2:** Baseline patient characteristics

Characteristics	*N* = 12
Demographics	
Age, years	58.5 +/− 8.5
Female, *n* (%)	3 (25)
Education, *n* (%)	
Bachelor degree or higher	9 (75)
Advanced diploma/diploma	2 (17)
School certificate	1 (8)
Employment, *n* (%)	
Full time employment	8 (67)
Employment part-time/casual	1 (8)
Retired/pensioner	2 (17)
Other	1 (8)
Current/former smoker, *n* (%)	4 (33)
Clinical characteristics	
Body mass index, kg/m^2^	31 +/−5.7
Systolic blood pressure, mmHg	136.3 +/−14.1
Diastolic blood pressure, mmHg	81.9 +/− 7.9
HbA1c, %^a^	7.1 +/− 0.9
History of hypertension, *n* (%)	9 (75)
History of diabetes, *n* (%)	9 (75)

Mean +/− SD unless otherwise indicated

^a^Data available in 7 patients

**Fig. 2:**
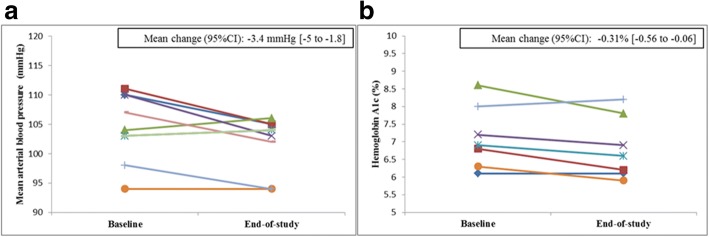
**a** Mean arterial blood pressure in patients who received wireless blood pressure home monitoring. Data collected automatically via the monitors. **b** Hemoglobin A1c ascertained from available data in electronic health records collected during routine clinical care

All patients received PTMs with a total of 454 messages sent; 95 were bidirectional. Patients responded to 50% of the bidirectional texts. Most patients reported that PTMs were easy to understand (88%), and appropriate in frequency (71%) and language (88%). We collected 77% of expected BP recordings. Nine participants with a history of hypertension received the wireless BP home monitoring. All of them reported the BP monitoring to be useful, and nearly all (89%) found it easy to use. At the end of the study, 82% of participants reported high medication adherence vs. 75% at baseline. After review of BP results at the mid-point, a change in anti-hypertensive therapy was made in 4 out of 9 patients (3 changed prescriptions; 1 reduced salt intake). Mean BP improved from 147/84 mmHg (week 0) to 143/81 mmHg (week 4–7). Mean arterial pressure was lower at the end-of-study compared to baseline (− 3.4 mmHg [95% CI, − 5 to − 1.8]; Fig. 2a). In patients with available data (*n* = 7), HbA1c was lower in most patients (Fig. 2b) compared to baseline. Mean change in HbA1c among all patients was − 0.31% [95% CI, − 0.56 to − 0.06].

## Discussion

This pilot study provided valuable data to improve and redefine study procedures (such as recruitment method, text messages bank, cloud-based portal) for a future full-scale study.

### Principal findings

This pilot study found that in patients with cardiometabolic disease, defined as type 2 diabetes and/or hypertension, the concomitant application of wireless BP monitoring and semi-personalized text messages based on the behavioral economic principles of automated hovering and nudging was feasible and acceptable. In patients with a wide age range and broad educational backgrounds, this pilot study demonstrates the feasibility and acceptability of a practical intervention strategy that capitalizes on the availability of simple wireless technology to implement the emerging science of behavioral economics to guide patient-tailored behavior change.

Results from a U.S. survey reported that the majority of people across all income groups possess a mobile phone [18] and text messages are low cost and can be easily automated. Recent evidence supports the effectiveness of mobile phone text message-based interventions to influence lifestyle and health-related behaviors, such as exercise [19], weight loss, and medication adherence [20], in patients with chronic diseases. In our pilot study, participants reported an increase in medication adherence at the end of the study.

When compared to usual care, text messaging interventions [21] and BP monitoring [22] alone showed a small effect on BP, whereas a greater reduction in BP was observed after a combined intervention of BP monitoring and lifestyle counseling [23]. In our pilot study, we observed an improvement of mean arterial BP after a combined intervention of PTMs and wireless BP home monitoring, supporting the hypothesis of a positive synergic effect of such a concomitant intervention.

Our results are consistent with those of a RCT conducted among patients with coronary heart disease [24] where there was a greater improvement of systolic BP, BMI, physical activity and smoking status after a 6-month intervention of lifestyle-focused semi-personalized text messages [24].

In seven patients with available data, we observed a reduction of the HbA1c at the end of the study. This finding is in accordance with results from a recent RCT conducted among Hispanic patients with type 2 diabetes reporting a significant reduction of the HbA1c after a 6-month text message-based interventions using motivational, educational, and/or call-to-action text messages [25].

### Limitations

The study’s limitations (small number of patients and the lack of a control group) are consistent with the pilot nature of the study, whose main goal was to evaluate feasibility and acceptability and refine critical aspects of the overall approach [26]. Another limitation was that it was unclear to the investigators whether patients were receiving the text messages; therefore, a future study would send a bi-directional text at baseline asking patients to respond with a “yes” to indicate receipt. We also found that some older patients required additional training with the use of the BP home monitoring device.

## Conclusions

Although behavioral economics and wireless technology have separately shown promise for improving care of chronic conditions in the short term, the concurrent application of strategies identified by the field of behavioral economics (e.g., “automated hovering”, “nudging”) and tools from wireless technology (e.g., monitoring, texting) has not been tried previously in patients with type 2 diabetes and/or hypertension in a real-world health care setting. Our pilot study showed that this approach is feasible and acceptable to patients and we expect that such a multi-component intervention to have synergistic benefits; however, larger studies are needed to confirm effectiveness.

## Abbreviations

BP: Blood pressure
HbA1c: Hemoglobin A1c
IRB: Institutional review board
PTMs: Personalized text messages
